# Magnetic P(AA-AM)/SA-BC-Fe_3_O_4_ Composite Hydrogel: Synthesis, Characterization, and Enhanced Adsorption Performance for Methylene Blue

**DOI:** 10.3390/gels12050428

**Published:** 2026-05-13

**Authors:** Yunxiang Zheng, Yonghan Wang, Mengmeng Wang, Chunxiao Zhang, Xiangpeng Wang

**Affiliations:** 1 School of Chemical Engineering, Shandong Institute of Petroleum and Chemical Technology, Dongying 257061, China; zhengyunxiang1990@163.com (Y.Z.); 15166282528@163.com (Y.W.); 2 Shandong Key Laboratory of Green Electricity & Hydrogen Science and Technology, Shandong Institute of Petroleum and Chemical Technology, Dongying 257061, China; zcx@sdipct.edu.cn; 3 PetroChina West–East Gas Pipeline Company, Shanghai 200120, China; wangmm03@pipechina.com.cn; 4 College of Chemical Engineering and Environment, China University of Petroleum, Beijing 102249, China

**Keywords:** magnetic composite hydrogel, methylene blue adsorption, water treatment, biochar

## Abstract

The development of adsorbents with high adsorption capacity, easy separation, and good reusability is critical for the treatment of dye-contaminated wastewater. Herein, a novel magnetic composite hydrogel, P(AA-AM)/SA-BC-Fe_3_O_4_, was synthesized via free radical polymerization, integrating acrylic acid (AA), acrylamide (AM), sodium alginate (SA), biochar (BC), and magnetic Fe_3_O_4_ nanoparticles. The material was systematically characterized by FTIR, XRD, SEM, BET, and VSM, which confirmed the successful formation of a three-dimensional porous network with well-dispersed Fe_3_O_4_ nanoparticles and BC, endowing the hydrogel with superparamagnetic properties. The adsorption performance of the hydrogel towards methylene blue (MB) was evaluated under various conditions. The results demonstrated that the adsorption process followed the pseudo-second-order kinetic model and the Langmuir isotherm, indicating that chemisorption is an important mechanism in the monolayer adsorption process. The hydrogel exhibited excellent swelling properties and remarkable pH-dependent adsorption behavior, with optimal performance in weakly alkaline environments. Notably, the incorporation of BC enhanced the adsorption capacity, while Fe_3_O_4_ enabled rapid magnetic separation, with the adsorbent retaining approximately 77% of its initial capacity after five regeneration cycles. This work presents a promising strategy for constructing magnetic hydrogel adsorbents that synergistically combine high adsorption efficiency, facile separability, and good reusability for practical wastewater treatment applications.

## 1. Introduction

Water pollution, particularly from synthetic dyes such as MB, has become a pressing global environmental concern due to its adverse effects on aquatic ecosystems and human health [1]. Among various treatment technologies, adsorption has emerged as a highly effective and economically viable approach for dye removal from wastewater [2]. Hydrogels, three-dimensional crosslinked hydrophilic polymer networks, have attracted considerable attention as adsorbents owing to their high water content, tunable functional groups, and excellent biocompatibility [3]. In particular, hydrogels based on P(AA-AM) and SA offer abundant carboxyl and hydroxyl groups that serve as efficient adsorption sites for cationic dyes [4]. However, conventional hydrogels often suffer from limited mechanical strength, poor reusability, and difficulty in separation from aqueous solutions after use.

In our previous work, we developed a magnetic superabsorbent composite based on P(AA-AM)-g-SA/Fe_3_O_4_, which exhibited excellent water absorption and rapid magnetic separability [5], but its application for dye adsorption was not explored. To address the limitations of conventional hydrogels and to extend the functionality of our earlier system, the incorporation of functional fillers such as biochar and magnetic nanoparticles has been proposed as a promising strategy. Biochar, a low-cost carbonaceous material, can increase specific surface area and introduce additional adsorption sites, thereby improving the overall adsorption capacity [6]. Meanwhile, the introduction of magnetic Fe_3_O_4_ nanoparticles enables the adsorbent to be rapidly separated from the reaction medium under an external magnetic field, overcoming the separation bottleneck associated with conventional hydrogel adsorbents [7].

Although previous studies have explored either BC-reinforced hydrogels or magnetic hydrogels separately, the synergistic combination of BC and Fe_3_O_4_ within a P(AA-AM)/SA matrix remains underexplored. For instance, some reported BC-based hydrogels for MB adsorption did not incorporate magnetic components, making post-adsorption separation difficult [8]. Other magnetic alginate composites lacked BC, resulting in relatively low specific surface area and adsorption site density [9]. In comparison, the present ternary system (P(AA-AM)/SA-BC-Fe_3_O_4_) is designed to combine the high swellability and abundant functional groups of the polymer matrix, the enhanced absorption sites of BC, and the magnetic responsiveness of Fe_3_O_4_. To date, a systematic investigation into the interplay among these three components and their impact on MB adsorption performance, magnetic responsiveness, and reusability is still lacking. Moreover, most existing studies have focused on dye systems in distilled water without considering the effect of water matrix composition, which is critical for real wastewater treatment.

In this study, we report the synthesis and characterization of a novel magnetic composite hydrogel, P(AA-AM)/SA-BC-Fe_3_O_4_, via a one-pot free radical polymerization method. Compared with our previous material, the addition of biochar in the present work specifically targets enhanced MB adsorption through additional hydrophobic and electrostatic interactions, while the magnetic Fe_3_O_4_ ensures easy recovery. The preparation conditions were systematically optimized, and the physicochemical properties of the hydrogel were comprehensively characterized. The adsorption performance of the composite toward MB was evaluated under various conditions, including initial dye concentration, pH, adsorbent dosage, and water matrix composition. Furthermore, the adsorption kinetics, isotherms, and underlying mechanisms were elucidated through model fitting and spectroscopic analysis. This work provides a comprehensive understanding of the structure-property-performance relationships of the ternary composite hydrogel, highlighting its potential as a high-performance, magnetically separable adsorbent for efficient dye removal from complex wastewater environments.

## 2. Results and Discussion

### 2.1. Structural Characterization

**Figure 1:**
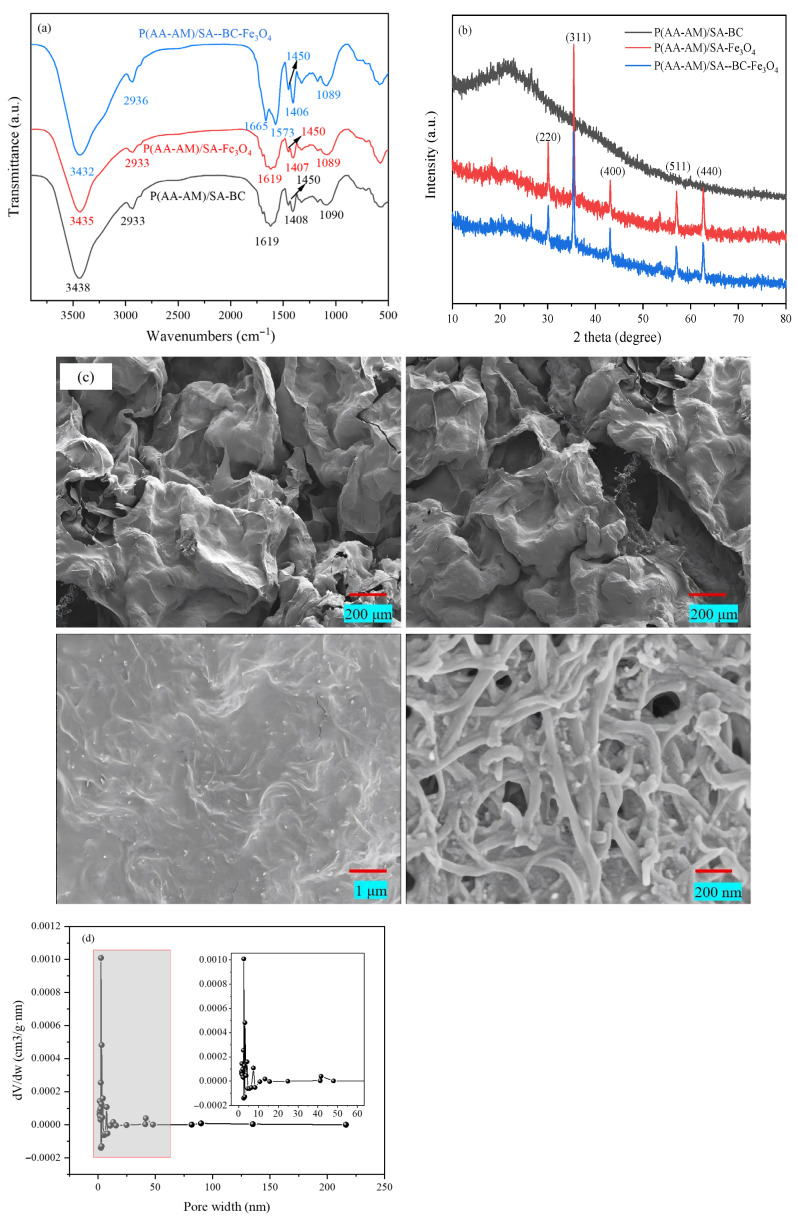
(**a**) FTIR spectra, (**b**) XRD patterns, (**c**) SEM images of the P(AA-AM)/SA-BC-Fe_3_O_4_, and pore width distribution of the P(AA-AM)/SA-BC-Fe_3_O_4_, (**d**) pore size distribution.

The FTIR spectra of the three hydrogels are shown in Figure 1a. The absorption bands at 3438–3432 cm^−1^ and 2933–2936 cm^−1^ correspond to O–H stretching and C–H stretching of methyl/methylene groups, respectively [10]. In the region of 1619–1665 cm^−1^ and 1573 cm^−1^, both P(AA-AM)/SA-BC and P(AA-AM)/SA-Fe_3_O_4_ exhibit characteristic absorptions of amide C=O or carboxylate groups. Notably, the ternary composite displays two distinct peaks at 1665 cm^−1^ and 1573 cm^−1^, whereas the binary systems show only a single peak at 1619 cm^−1^. The bands at 1450 cm^−1^ and 1408–1406 cm^−1^ are attributed to symmetric stretching vibrations of carboxyl groups. All samples show a characteristic peak at 1090–1089 cm^−1^ corresponding to C–O–C or C–OH stretching, indicating the intact polymer backbone [11]. Collectively, the systematic shifts and splitting of characteristic IR peaks confirm the successful incorporation of biochar and Fe_3_O_4_, with the ternary system exhibiting a more complex interaction network involving hydrogen bonding and electrostatic interactions.

Figure 1b shows the XRD patterns of the three composite hydrogels. P(AA-AM)/SA-BC exhibits a broad and diffuse peak in the 2θ range of 10–30°, originating from the combined contribution of the amorphous polymer matrix and the amorphous structure of biochar, indicating a predominantly non-crystalline nature with extremely low crystallinity. P(AA-AM)/SA-Fe_3_O_4_ displays characteristic diffraction peaks at approximately 30.1°, 35.5°, 43.1°, 57.0°, and 62.6°, corresponding to the (220), (311), (400), (511), and (440) crystal planes of Fe_3_O_4_, respectively [12]. These peaks are in full agreement with the standard XRD pattern of Fe_3_O_4_, confirming the successful incorporation of Fe_3_O_4_ nanoparticles into the hydrogel network while preserving their crystalline structure. The pattern of P(AA-AM)/SA-BC-Fe_3_O_4_ shows diffraction peaks consistent with those of P(AA-AM)/SA-Fe_3_O_4_, clearly displaying the characteristic Fe_3_O_4_ crystal planes alongside the broad amorphous hump from the polymer and biochar. This indicates that the crystal structure of Fe_3_O_4_ remains intact after biochar introduction, with all components achieving good structural coexistence in the composite hydrogel.

Figure 1c shows the SEM images of P(AA-AM)/SA-BC-Fe_3_O_4_ at different magnifications. At low magnification (200 μm scale bar), the hydrogel exhibits a typical three-dimensional, wrinkled, porous macrostructure, with lamellae stacked and interconnected to form continuous open pores. This loose network provides ample space for water diffusion and solute transport. At medium magnification (1 μm scale bar), finer textures are observed on the wrinkled lamellar surfaces, where biochar and the polymer matrix show tight interfacial adhesion with no obvious phase separation, indicating relatively uniform dispersion of the components within the hydrogel network. At high magnification (200 nm scale bar), Fe_3_O_4_ nanoparticles are relatively uniformly distributed on the lamellar surfaces and within the skeleton, either attached or embedded, further confirming good dispersibility of Fe_3_O_4_ in the composite system. Meanwhile, nanoscale pores and fibrous structures between the lamellae are clearly visible, suggesting that this hierarchical porous structure synergistically enhances the specific surface area and adsorption/mass transfer efficiency of the material. The pore size distribution in Figure 1d reveals that P(AA-AM)/SA-BC-Fe_3_O_4_ features a primarily mesoporous architecture, with the majority of pores in the mesopore range and a mean diameter of 22.49 nm. Consequently, these mesopores can serve as conduits for the transport of MB molecules.

### 2.2. Magnetic Properties

**Figure 2:**
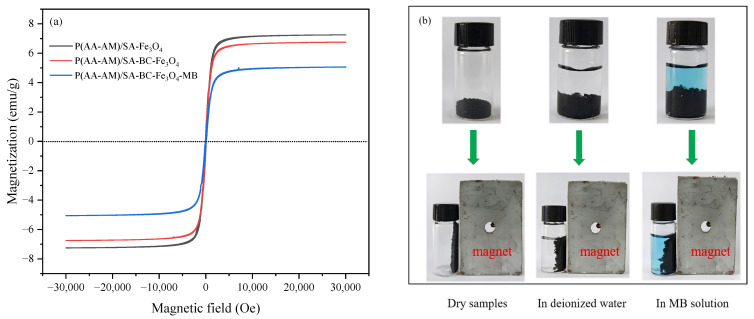
(**a**) Magnetization curves; (**b**) magnetic property photograph of P(AA-AM)/SA-BC-Fe_3_O_4_.

As shown in Figure 2a, all samples exhibit typical S-shaped curves with no hysteresis loop, showing no remanence or coercivity within the ±30,000 Oe magnetic field range, confirming the superparamagnetic nature of Fe_3_O_4_ nanoparticles embedded in the hydrogel matrix. This characteristic ensures no magnetic residue in the absence of an external field, preventing particle aggregation and structural agglomeration. Among the samples, P(AA-AM)/SA-Fe_3_O_4_ shows the highest saturation magnetization of approximately 7.2 emu/g. After biochar incorporation, the magnetization of P(AA-AM)/SA-BC-Fe_3_O_4_ slightly decreases to about 6.8 emu/g, attributable to the dilution effect of the non-magnetic biochar component. Following MB adsorption, the magnetization of P(AA-AM)/SA-BC-Fe_3_O_4_-MB further declines to approximately 5.1 emu/g, indicating that adsorption of organic dye molecules partially shields the magnetic response of Fe_3_O_4_ while still maintaining sufficient magnetic separability.

The magnetic response photographs in Figure 2b visually confirm the magnetic separation performance. The composite hydrogels—whether in the dry state, dispersed in deionized water, or in MB solution—can be rapidly and directionally moved and aggregated under an external magnetic field, enabling efficient solid–liquid separation even in aqueous environments. This provides key technical support for their recovery and reuse in practical applications such as wastewater treatment. Collectively, the incorporation of Fe_3_O_4_ endows the composite hydrogels with excellent superparamagnetism and magnetic responsiveness. The introduction of biochar and dye adsorption only moderately reduces the saturation magnetization without compromising magnetic separation capability, demonstrating the feasibility and stability of this ternary composite hydrogel for coupled adsorption-magnetic separation applications. It should be noted, however, that after repeated adsorption cycles, the magnetic separation rate does decrease to some extent due to the gradual reduction in magnetization. Nevertheless, under the same external magnetic field strength, the majority of the hydrogel can still be effectively separated from the solution. In subsequent cycles, the separation efficiency can be restored or improved by appropriately increasing the magnetic field strength to compensate for the loss in magnetization.

### 2.3. Optimization of Preparation Conditions for P(AA-AM)/SA Hydrogel

**Figure 3:**
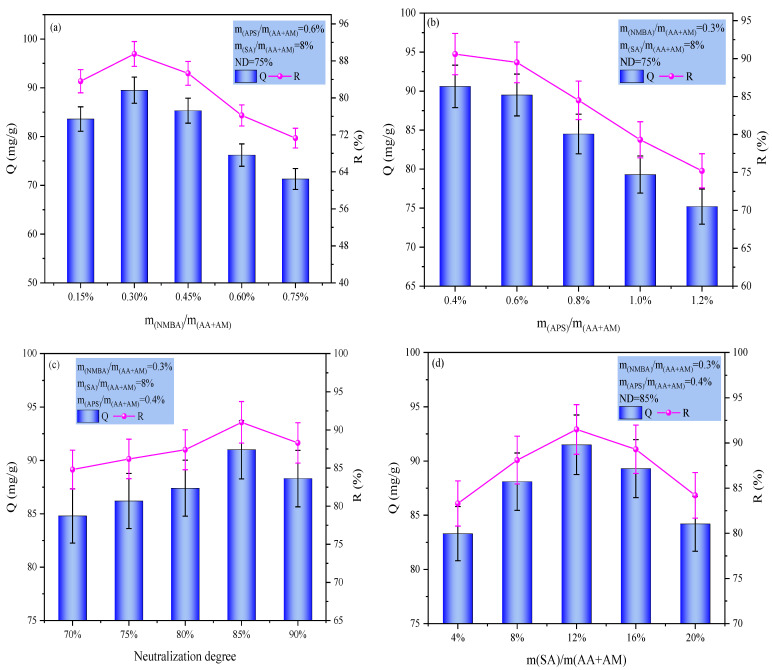
Optimization of polymerization conditions: (**a**) NMBA; (**b**) APS; (**c**) ND; and (**d**) SA.

This study systematically investigated the effects of NMBA, APS, neutralization degree (ND) of acrylic acid, and SA dosage on the MB adsorption performance of P(AA-AM)/SA hydrogels. As shown in Figure 3, with increasing NMBA dosage, the adsorption performance gradually increased and reached a peak, beyond which further increase led to a significant decline in adsorption capacity. This is attributed to the loose network structure and susceptibility to swelling-induced mass loss at excessively low crosslinking densities (a mass loss of ~3.6% was measured after swelling and re-drying at an NMBA loading of 0.15%), whereas excessive crosslinking renders the pore structure overly dense, hindering the diffusion and accessibility of methylene blue molecules [13]. From a mechanistic perspective, the crosslinker concentration directly determines the number of effective crosslinking points, which governs both the equilibrium swelling ratio and the diffusional permeability of the gel network.

Excessive APS initiator dosage tends to cause overly rapid polymerization and a high concentration of free radicals, which promotes chain transfer and termination reactions, resulting in heterogeneous crosslinking structures and non-uniform network architecture—both of which compromise adsorption performance.

The ND of acrylic acid directly determines the charge state and swelling capacity of the hydrogel. At low neutralization degrees, insufficient dissociation of carboxyl groups results in contracted polymer chains and fewer negatively charged sites. As the ND increases, the progressive deprotonation of –COOH groups introduces negative charges along the polymer backbone. The resulting electrostatic repulsion between polymer chains promotes chain expansion and network enlargement, thereby increasing the free volume and exposing more active sites for dye binding, substantially improving adsorption performance. However, excessive neutralization leads to a salting-out effect due to elevated ionic strength within the system, simultaneously reducing swelling and adsorption capacities [14].

SA forms a semi-interpenetrating network with the polymer matrix; its abundant hydroxyl and carboxyl groups provide additional adsorption sites and act as physical entanglement points that optimize the pore structure and enhance mechanical stability. At an appropriate dosage, the SA chains interpenetrate the synthetic network without significant phase separation, creating a more open and interconnected porous architecture. Excess SA significantly increases system viscosity, disrupts copolymerization uniformity, and induces phase separation, resulting in disordered pore structures and hindered mass transfer, ultimately diminishing adsorption performance [15].

In summary, under the optimal conditions where NMBA, APS, and SA dosages were 0.3%, 0.4%, and 12% of the total monomer mass, respectively, with an ND of 85%, the hydrogel exhibited the optimal adsorption capacity (91.5 mg/g) and removal efficiency (91.5%) for MB. Subsequent investigations into the effects of biochar and Fe_3_O_4_ dosages on the adsorption performance of the composite hydrogels were conducted under these conditions.

### 2.4. Effect of BC and Fe_3_O_4_ Dosages on Adsorption Performance

**Figure 4:**
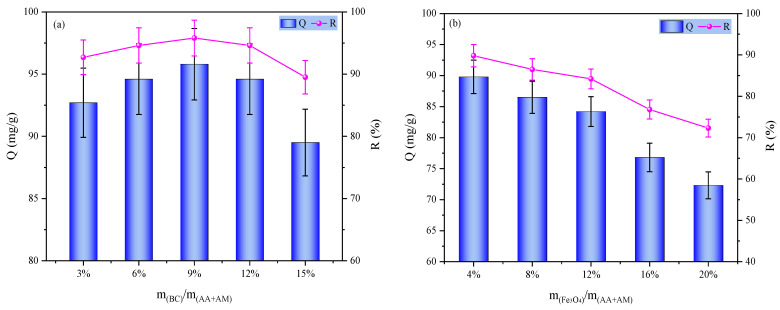
Effect of (**a**) BC and (**b**) Fe_3_O_4_ dosages on adsorption performance.

Based on the optimized preparation of P(AA-AM)/SA hydrogels, the effects of BC and Fe_3_O_4_ dosages on the adsorption performance for MB were further investigated. As shown in Figure 4a, with increasing BC dosage, the adsorption capacity of the hydrogels continuously increased, reaching a peak value of 95.8 mg/g at a BC dosage of 9%. Further increasing the BC dosage to 15% led to a significant decline in adsorption performance. This is because an appropriate amount of BC introduces a hierarchical pore structure; its abundant pores and surface oxygen-containing functional groups provide additional adsorption sites for MB, while synergistically interacting with the polymer network to enhance the specific surface area and adsorption affinity. However, excessive BC disrupts the uniformity of the hydrogel network and causes pore blockage, hindering the diffusion and mass transfer of MB molecules, ultimately weakening the adsorption performance [16].

As the Fe_3_O_4_ content increased from 4% to 20%, the adsorption performance exhibited a monotonic decreasing trend (Figure 4b). This is attributed to the fact that Fe_3_O_4_ is a non-adsorptive magnetic component; although its incorporation endows the hydrogel with magnetic separation functionality, it occupies a portion of the adsorption sites and blocks the pore channels. As the Fe_3_O_4_ dosage increases, this dilution and blockage effect becomes more pronounced, leading to a reduction in effective adsorption sites and mass transfer efficiency, thereby continuously diminishing the MB adsorption capacity.

Overall, the addition of BC and Fe_3_O_4_ involves a significant performance trade-off: an appropriate amount of BC enhances adsorption performance, whereas the incorporation of Fe_3_O_4_ improves magnetic responsiveness at the expense of a partial reduction in adsorption capacity.

**Figure 5:**
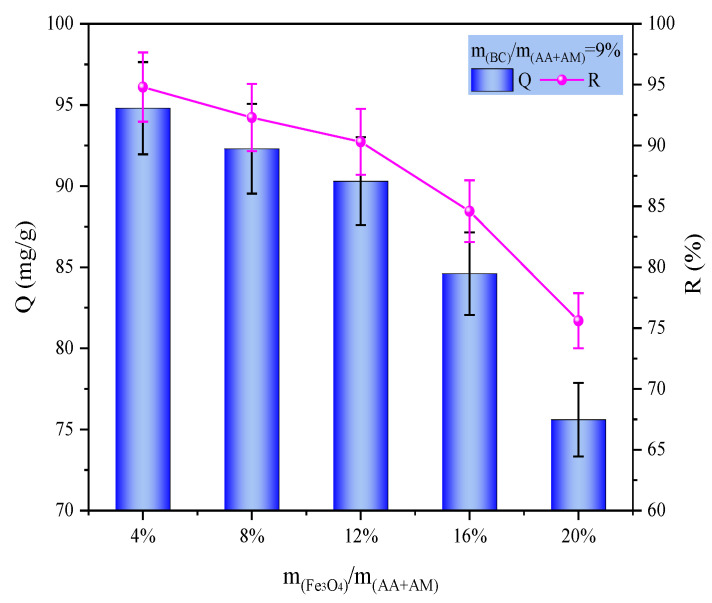
Effect of Fe_3_O_4_ dosages on P(AA-AM)/SA-BC-Fe_3_O_4_ MB adsorption performance.

Therefore, under the condition of a BC addition of 9%, we further investigated the effect of Fe_3_O_4_ dosage on the MB adsorption performance of the composite hydrogel. The results are shown in Figure 5. As the Fe_3_O_4_ dosage increased, the adsorption capacity of the hydrogel gradually decreased. To balance the dye-removal performance and magnetic properties of the hydrogel, we ultimately chose to prepare the hydrogel at an Fe_3_O_4_ mass fraction of 12% relative to the monomers and used this formulation to evaluate its comprehensive performance. All subsequent investigations on the hydrogel properties were conducted under this condition.

### 2.5. Swelling Properties of P(AA-AM)/SA-BC-Fe_3_O_4_

**Figure 6:**
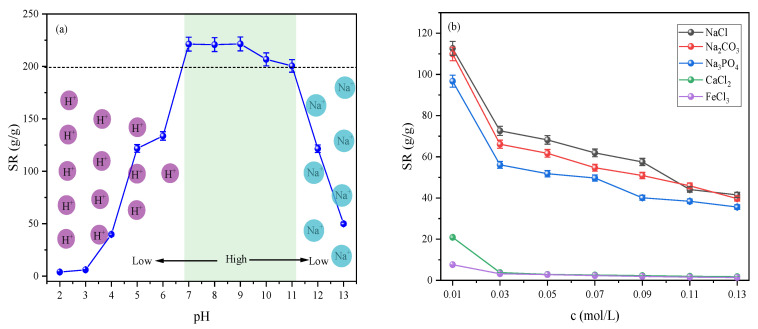
Swelling behavior of P(AA-AM)/SA-BC-Fe_3_O_4_ composite hydrogel under different (**a**) pH and (**b**) salt solution conditions.

Figure 6a,b systematically illustrate the swelling behavior of the P(AA-AM)/SA-BC-Fe_3_O_4_ composite hydrogel under varying pH and salt solution environments. As shown in Figure 6a, the swelling ratio (SR) of the hydrogel exhibited a U-shaped trend with changing pH, reaching a peak value in the pH range of 7–11. Under strongly acidic conditions (pH < 5), carboxyl groups were protonated, significantly weakening interchain electrostatic repulsion, while H^+^ formed hydrogen bonds with the network, leading to polymer chain contraction and collapse, resulting in a low swelling ratio. As the pH increased to the neutral to weakly alkaline range, carboxyl groups became fully dissociated into –COO^−^, and the enhanced electrostatic repulsion between chains promoted full expansion of the three-dimensional network, substantially improving swelling performance. In strongly alkaline environments (pH > 11), the salt effect induced by excess Na^+^ compressed the electrical double layer, weakening interchain electrostatic repulsion and causing a sharp decline in the swelling ratio [17]. Figure 6b shows that the SR of the hydrogel decreased with increasing salt concentration across various salt solutions, with significant differences observed depending on ion valence and type. Within the Flory–Rehner–Donnan (FRD) framework for ionic hydrogels, the equilibrium swelling is governed by the balance of mixing free energy, elastic free energy, and ionic free energy (electrostatic repulsion among fixed charges and Donnan osmotic pressure). Among monovalent sodium salts (NaCl, Na_2_CO_3_, Na_3_PO_4_), higher valence anions exerted a more pronounced inhibitory effect on swelling due to stronger charge screening, which reduces the ionic free energy contribution by collapsing the Donnan potential. Divalent Ca^2+^ and trivalent Fe^3+^ formed ionic crosslinking “bridges” with carboxyl groups, causing rapid network contraction even at very low concentrations and reducing the swelling ratio to near zero, highlighting a clear ion valence effect [18]. These observations are consistent with the FRD prediction that external cations screen anionic carboxylate groups (–COO^−^), thereby diminishing electrostatic repulsion and shifting the swelling equilibrium toward a lower swelling ratio.

In summary, this composite hydrogel achieved efficient swelling under neutral to weakly alkaline conditions and low salt concentrations, whereas swelling performance was significantly constrained in high-concentration or high-valence ion systems. This behavior provides important structural and performance insights for selecting operating conditions in practical wastewater treatment scenarios and adsorption applications.

### 2.6. MB Adsorption Performance of P(AA-AM)/SA-BC-Fe_3_O_4_ Composite Hydrogel

**Figure 7:**
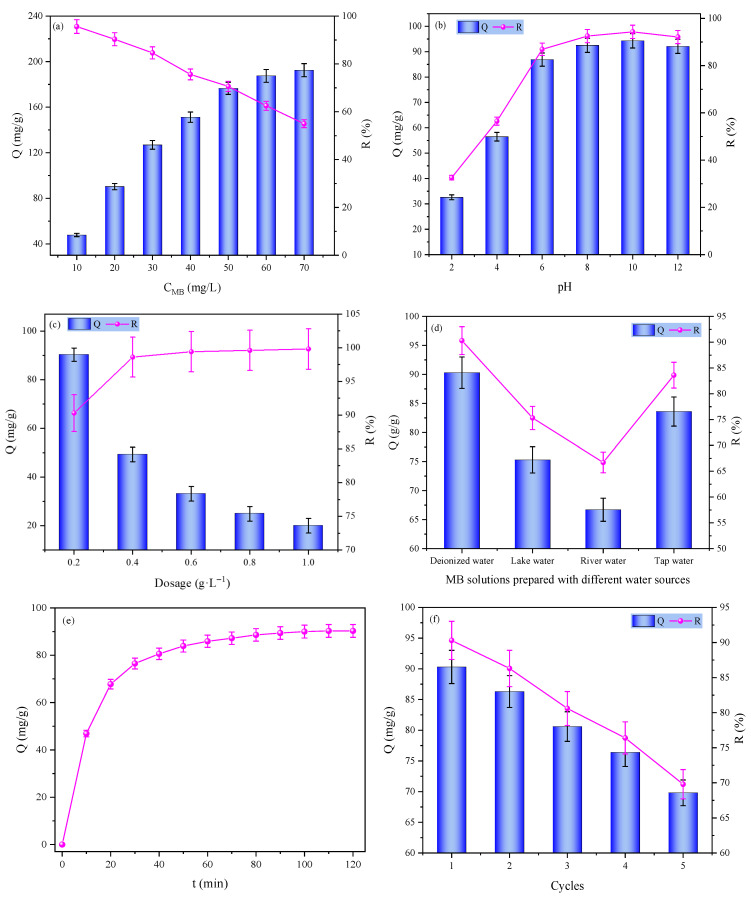
Adsorption performances of P(AA-AM)/SA-BC-Fe_3_O_4_ hydrogel: (**a**) initial MB concentration; (**b**) solution pH; (**c**) adsorbent dosage; (**d**) water matrix; (**e**) adsorption kinetics; (**f**) reusability.

Figure 7a illustrates the influence of initial MB concentration on the adsorption performance of the hydrogel. As the initial MB concentration increased from 10 to 70 mg/L, the removal efficiency declined gradually from 95.6% to 55%. At low concentrations, abundant adsorption sites ensure nearly complete MB removal, yet the unit adsorption capacity is limited by the weak driving force from the concentration gradient. With increasing initial MB concentration, the enhanced concentration gradient drives greater diffusion of MB molecules toward the hydrogel surface, significantly boosting the unit adsorption capacity. However, the progressive saturation of adsorption sites at high concentrations leads to a higher proportion of unadsorbed MB, resulting in a continuous decrease in removal efficiency [19]. These results confirm the hydrogel’s potential for treating high-load dye wastewater.

Figure 7b demonstrates the pH-dependent adsorption behavior of the hydrogel toward MB. Within the pH range of 2–12, both Q and R increased initially and then stabilized, reaching a maximum at pH of 10 (Q = 94.3 mg/g, R = 94.3%). This behavior is governed by the polyelectrolyte nature of the hydrogel: under strongly acidic conditions, carboxyl groups (–COO^−^) are protonated to –COOH, reducing the density of negative charge sites and inhibiting electrostatic attraction with cationic MB. As pH rises into the weakly alkaline range, carboxyl groups fully dissociate, maximizing negative charge density and strengthening electrostatic interactions, while avoiding the salt-induced compression of the electrical double layer that occurs at high pH [20]. Further increasing pH to strongly alkaline conditions leads to a slight decline in adsorption performance due to charge screening by excess Na^+^ ions. These results verify that a weakly alkaline environment is optimal for the hydrogel’s adsorption activity.

Figure 7c illustrates the effect of adsorbent dosage on the Q and R of MB. As the adsorbent dosage increased from 0.2 g·L^−1^ to 1.0 g·L^−1^, the R monotonically increased from 90.3% to 99.8%, which can be attributed to the availability of more adsorption sites, leading to a lower residual dye concentration in the solution. In contrast, the unit adsorption capacity Q significantly decreased from 90.3 mg/g to 19.9 mg/g. This trend is consistent with the classical adsorption equilibrium law: under fixed initial dye concentration and solution volume, increasing the adsorbent dosage reduces the amount of dye allocated per unit mass of adsorbent, thereby lowering the equilibrium adsorption capacity. This behavior aligns with the prediction of the Langmuir model, which assumes homogeneous adsorption sites and a fixed saturation capacity; thus, excessive adsorbent dosage leads to a decrease in unit adsorption capacity.

Figure 7d evaluates the hydrogel’s adsorption performance in different water matrices, revealing that Q and R follow the order: deionized water > tap water > lake water > river water. This discrepancy arises from the interference of coexisting components in natural waters: deionized water contains no competing ions, allowing unimpeded adsorption. In contrast, lake and river waters contain high concentrations of divalent/polyvalent cations and natural organic matter. These species not only compete with MB for adsorption sites but also induce ionic cross-linking, compressing the electrical double layer and weakening electrostatic interactions. Additionally, natural organic matter may block hydrogel pores, further reducing mass transfer efficiency. The electrical conductivity of lake water and river water was measured at 1096 µS/cm and 4740 µS/cm, respectively, providing a quantitative indicator of their ionic strength. The higher electrical conductivity of river water corresponds to its lower adsorption performance. These findings demonstrate the hydrogel’s practical applicability in complex water matrices and provide guidance for pretreatment strategies.

Figure 7e displays the adsorption kinetic curve of MB onto the hydrogel, exhibiting a typical “fast adsorption-slow equilibrium” pattern: rapid adsorption occurs within 0–60 min, followed by a gradual slowdown until equilibrium is achieved at 120 min. This process can be divided into two stages: the initial stage is dominated by fast external diffusion and site occupation, driven by a high MB concentration gradient and abundant surface sites. In the later stage, adsorption is rate-limited by intraparticle diffusion and site saturation, as the concentration gradient diminishes and available sites decrease. This kinetic behavior indicates the hydrogel’s rapid response to MB contamination, making it suitable for continuous-flow or rapid-treatment scenarios.

Figure 7f presents the hydrogel’s reusability over five adsorption–desorption cycles. The initial Q and R were 90.3 mg/g and 90.3%, respectively, and after five cycles, Q decreased to 69.8 mg/g and R to 69.8%, retaining ~77% of the initial adsorption capacity. The performance decay is attributed to two main factors: (1) partial irreversible binding of MB molecules to adsorption sites, preventing full regeneration; (2) minor structural damage to the hydrogel network (e.g., pore collapse, chain scission) during repeated cycles, reducing specific surface area and mass transfer efficiency. The proposed degradation mechanisms—irreversible site blocking and network structural damage—are consistent with those widely reported for analogous hydrogel adsorbents and observed in our previous studies [21,22]. Notably, the neutralization degree adopted in this study exacerbates such structural damage, as the highly ionized carboxyl groups lead to excessive swelling and reduced mechanical stability under cyclic swelling–shrinking stresses. Consequently, the network becomes more susceptible to fatigue-induced pore collapse and chain scission, which is consistent with the observed decline in removal efficiency from 90.3% to 69.8% after five cycles. Despite this attenuation, the hydrogel demonstrates promising reusability, which is critical for reducing operational costs and enabling sustainable material reuse in practical applications.

### 2.7. Adsorption Kinetics, Isotherms, and Mechanisms

**Figure 8:**
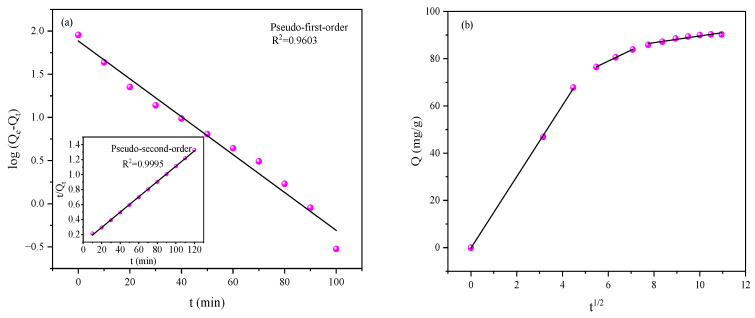
Kinetic analysis of MB adsorption: (**a**) pseudo-first-order and pseudo-second-order model fittings; (**b**) intra-particle diffusion model fitting.

The adsorption kinetics of MB onto hydrogel were systematically investigated using pseudo-first-order (Equation (1)), pseudo-second-order (Equation (2)), and intra-particle diffusion (Equation (3)) models, with the fitting results presented in Figure 8a,b, and the corresponding parameters summarized in Table 1.

(1) $${\log(Q}_{e}-Q_{t})=-\frac{k_{1}}{2.303}t+{\mathrm{logQ}}_{e}$$

(2) $$\frac{t}{Q_{t}}=\frac{t}{Q_{e}}+\frac{1}{k_{2}}\frac{1}{{Q_{e}}^{2}}$$

(3) $$Q_{t}{=\;\mathrm{Kt}}^{\frac{1}{2}}+C$$

where Q_t_ refers to the adsorption amount of MB on the hydrogel at time t; Q_e_ corresponds to the equilibrium adsorption capacity at adsorption saturation; k_1_ and k_2_ represent the rate constants of the pseudo-first-order and pseudo-second-order kinetic models, respectively; K stands for the rate constant of the intra-particle diffusion process; and C is a constant related to the thickness and resistance of the boundary layer during mass transfer.

As shown in Figure 8a, the pseudo-second-order model yields a significantly higher correlation coefficient (R^2^ = 0.9995) compared to the pseudo-first-order model (R^2^ = 0.9603), and the calculated equilibrium adsorption capacity is highly consistent with the experimental value, which is often considered as an indication of chemisorption-dominated behavior. Nevertheless, this interpretation is primarily derived from kinetic fitting and should therefore be regarded as preliminary. This result suggests that the rate-limiting step involves chemical interactions (e.g., electrostatic attraction, hydrogen bonding) between the MB molecules and the functional groups on the hydrogel surface, rather than simple physical diffusion [23].

Figure 8b displays the intra-particle diffusion plot, which exhibits a multi-linear profile with three distinct stages, revealing that the adsorption process is controlled by multiple mass transfer steps. The first stage corresponds to the rapid external diffusion of MB molecules from the bulk solution to the hydrogel surface, driven by a high concentration gradient. The second stage reflects the gradual intra-particle diffusion of MB into the hydrogel’s porous network, where the rate is limited by pore diffusion and site availability. The third stage represents the final equilibrium phase, where adsorption is restricted by the saturation of active sites and the reduced concentration gradient [24]. The non-zero intercept of the linear segments further confirms that intra-particle diffusion is not the sole rate-limiting step, and that boundary-layer resistance also contributes to the overall adsorption kinetics.

**Figure 9:**
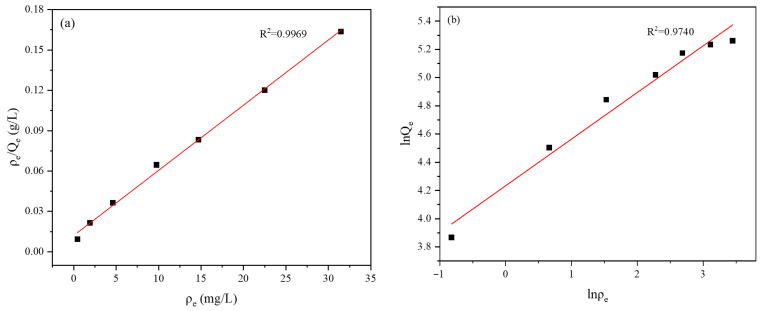
Adsorption isotherm fittings of MB onto hydrogel: (**a**) Langmuir model; (**b**) Freundlich model.

The Langmuir (Equation (4)) and the Freundlich (Equation (5)) isotherm models were employed to fit the equilibrium adsorption behavior of MB onto the hydrogel, and the results are presented in Figure 9. The Langmuir model exhibited a strong linear relationship (Figure 9a), with a correlation coefficient of 0.9969, significantly higher than that of the Freundlich model (Figure 9b). This indicates that the Langmuir model provides a more accurate description of the adsorption process, suggesting that the adsorption of MB onto the hydrogel surface occurs primarily via homogeneous monolayer coverage.

The superior goodness-of-fit of the Langmuir model further confirms the homogeneity of the adsorption sites, which is closely associated with the presence of functional groups such as carboxyl and hydroxyl moieties on the hydrogel surface [25]. In contrast, the relatively lower fitting degree of the Freundlich model suggests that the adsorption process does not follow a typical multilayer heterogeneous adsorption mechanism, thereby reinforcing the conclusion that monolayer adsorption is the dominant mode.

(4) $$\frac{\rho_{e}}{Q_{e}}=\frac{1}{Q_{0}K_{b}}+\frac{\rho_{e}}{Q_{0}}$$

(5) $${\mathrm{lnQ}}_{e}={\mathrm{lnK}}_{f}+\frac{1}{n}{\ln\rho }_{e}$$

where ρ_e_ stands for the residual MB concentration in solution following adsorption, mg/L. Q_e_ denotes the amount of MB adsorbed at equilibrium, mg/g. Q_0_ refers to the maximum adsorption capacity under theoretical conditions, mg/g. The constant K_b_ represents the adsorption equilibrium constant, L/mg. K_f_, another constant associated with the adsorption process, mg^1−n^·L^n^·g^−1^, while n is the dimensionless adsorption intensity factor.

**Figure 10:**
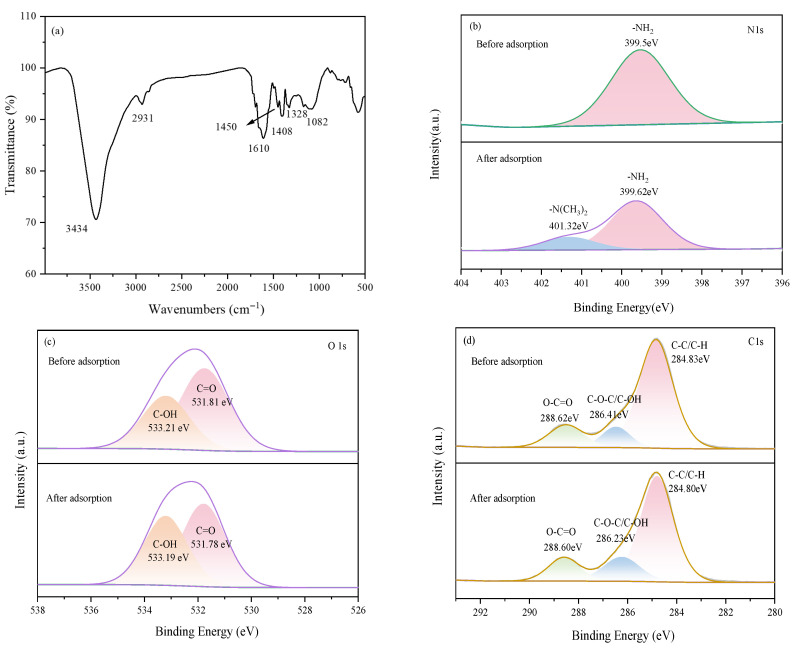
(**a**) FTIR spectrum of the hydrogel after MB adsorption, and XPS spectra before and after MB adsorption: (**b**) N1s spectra; (**c**) O1s spectra; (**d**) C1s spectra.

The infrared spectra of the hydrogel after methylene blue adsorption are shown in Figure 10a. Following adsorption, the stretching vibration peak of hydroxyl (–OH) and amino (–NH) groups, originally located at 3432 cm^−1^, blue-shifted to 3434 cm^−1^, indicating changes in electron cloud density due to hydrogen bonding interactions. In the carboxyl/amide region, the peaks at 1665 cm^−1^ (amide I band) and 1573 cm^−1^ (asymmetric stretching of carboxylate) merged into a strong, broad peak at 1610 cm^−1^ after adsorption, accompanied by a slight shift in the symmetric carboxylate stretching peak from 1406 cm^−1^ to 1408 cm^−1^. These significant changes confirm the occurrence of strong electrostatic interactions between carboxylate groups and MB cations, representing an important interaction mechanism for the adsorption process. Additionally, the C–O–C/C–OH stretching peak at 1089 cm^−1^ shifted to 1082 cm^−1^, further supporting hydrogen-bonding interactions between the polysaccharide backbone and the dye molecules [26]. The emergence of a new peak at 1328 cm^−1^ after adsorption corresponds to characteristic vibrations of the aromatic heterocyclic rings in MB, providing direct evidence for successful dye loading. The C–H bending vibration at 1450 cm^−1^ remained largely unchanged, indicating the structural integrity of the material framework.

By comparing the XPS spectra of the hydrogel before and after adsorption, the interaction mechanism with MB can be further elucidated. In the N1s spectrum (Figure 10b), in addition to the original –NH_2_ peak (shifted from 399.5 eV to 399.62 eV), a new characteristic peak corresponding to –N(CH_3_)_2_ appeared at 401.32 eV after adsorption, which directly corresponds to the dimethylamino group in MB molecules, confirming the successful loading of MB onto the hydrogel surface. In the O1s spectrum (Figure 10c), the C–OH (533.21 eV to 533.19 eV) and C=O (531.81 eV to 531.78 eV) peaks both exhibited slight shifts, indicating that hydroxyl and carbonyl functional groups participated in hydrogen bonding or electrostatic interactions with MB. In the C1s spectrum (Figure 10d), the peaks corresponding to C–C/C–H (284.83 eV to 284.80 eV), O–C=O (288.62 eV to 288.60 eV), and C–O–C/C–OH (286.41 eV to 286.23 eV) also showed subtle shifts, suggesting changes in electron cloud density around the carbon skeleton and oxygen-containing functional groups during adsorption, which further supports the occurrence of electrostatic attraction and hydrogen bonding as key contributing mechanisms [27]. Collectively, the XPS results, together with the FTIR analysis, clearly demonstrate that efficient adsorption is driven by the synergistic effects of electrostatic interactions and hydrogen bonding between the active sites (such as carboxyl and hydroxyl groups) on the hydrogel surface and MB molecules.

### 2.8. Comparison with Other Adsorbents

Based on the comparison of MB adsorption performance in Table 2, it is evident that the adsorption capacity (Q) is strongly influenced by experimental conditions such as initial concentration and pH. Under alkaline conditions (pH 10.0) and a relatively low initial concentration (20 mg/L), the hydrogel developed in this study achieves a Q value of 94.3 mg/g. This performance is superior to several previously reported biochar and hydrogel composites (e.g., N-doped biochar: 84.2 mg/g at 30 mg/L, pH 11.0 [28]; hydrogel-biochar composite: 23.53 mg/g at 20 mg/L, pH 11 [29]; HG/MTWBC nanocomposite: 20.79 mg/g at 10 mg/L, pH 8 [30]), and also outperforms a similar hydrogel-based adsorbent P(AA-AM)/SA-BC-Fe_3_O_4_ (this study) in terms of overall capacity under comparable conditions.

However, a more critical comparison reveals that some state-of-the-art adsorbents, such as β-cyclodextrin xanthate hydrogel/nickel oxide nanocomposites, exhibit a substantially higher Q (310.55 mg/g) under a much higher initial concentration (100 mg/L) and neutral pH [32]. This indicates that while our hydrogel shows good removal efficiency at low MB concentrations and alkaline conditions, its capacity at higher concentrations may be less competitive. Furthermore, differences in initial concentration and pH make direct capacity comparisons challenging. Therefore, rather than claiming general superiority, we conclude that the present hydrogel is a promising candidate for MB removal under specific conditions (low initial concentration, alkaline pH), but further optimization and evaluation under more realistic or variable conditions are needed to benchmark against the best-performing materials.

## 3. Conclusions

In this study, a magnetic composite hydrogel, P(AA-AM)/SA-BC-Fe_3_O_4_, was successfully synthesized via free radical polymerization and systematically evaluated for its adsorption performance toward MB. The preparation conditions, including crosslinker, initiator, neutralization degree of acrylic acid, and SA dosage, were optimized to achieve maximum adsorption capacity. The incorporation of BC introduced a hierarchical pore structure and additional functional groups, thereby enhancing adsorption capacity, while Fe_3_O_4_ nanoparticles endowed the hydrogel with superparamagnetic properties, enabling rapid magnetic separation from aqueous solutions. Structural characterization via FTIR, XRD, and SEM confirmed the successful integration of all components within a three-dimensional porous network with uniform dispersion of Fe_3_O_4_ and BC. The hydrogel exhibited notable swelling behavior, particularly under neutral to weakly alkaline conditions and low salt concentrations, and it showed considerable MB adsorption. The adsorption process followed pseudo-second-order kinetics and the Langmuir isotherm model, indicating a chemisorption-dominated monolayer adsorption mechanism. Mechanistic analysis revealed that electrostatic interactions and hydrogen bonding between the functional groups (carboxyl, hydroxyl) on the hydrogel and MB molecules were the primary driving forces for adsorption. Moreover, the hydrogel maintained reusability (retaining approximately 77% of its initial capacity after five adsorption–desorption cycles) and showed performance in several real water matrices under laboratory conditions, which suggests potential for further evaluation. Nevertheless, practical application in real-world wastewater treatment would require additional studies on long-term stability, competing substances, and scalability. Overall, this work presents a design strategy for developing magnetic hydrogel adsorbents, with the current results serving as a preliminary basis for future assessment in more complex treatment scenarios.

## 4. Materials and Methods

### 4.1. Materials

Acrylic acid (AA, 99%), acrylamide (AM, 98%), sodium alginate (SA, CP), N, N-methylenebisacrylamide (NMBA, 99%), ammonium persulfate (APS, 98%), sodium hydroxide (96%), hydrochloric acid (36.0%~38.0%), triiron tetraoxide (97%), methylene blue (MB, 98%), sodium chloride (99%), calcium chloride (98%), ferric chloride (97%), sodium carbonate (99%), sodium phosphate (96%) were purchased from Sinopharm Chemical Reagent Co., Ltd., Shanghai, China. Biochar (BC) was purchased from Henan Lize Environmental Protection Technology Co., Ltd., Zhengzhou, China. Deionized water, tap water, lake water (Qingfeng Lake, Dongying, China, electrical conductivity: 1096 µS/cm), and river water (Guangli River, Dongying, China, electrical conductivity: 4740 µS/cm) were used for the experiments.

### 4.2. Preparation of Magnetic P(AA-AM)/SA-BC-Fe_3_O_4_

**Figure 11:**
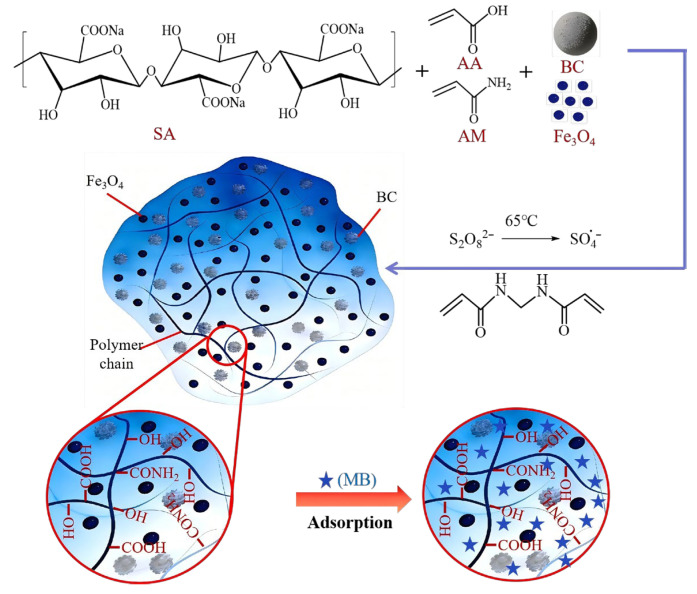
Schematic diagram of the preparation process of P(AA-AM)/SA-BC-Fe_3_O_4_.

Taking one polymerization reaction as an example: 85.0 g of deionized water was placed in a 250 mL beaker, and 2.3 g of SA was added. The mixture was magnetically stirred at room temperature until complete dissolution. Subsequently, 14.4 g of AA and 4.8 g of AM were added successively, and stirring was continued until the monomers were completely dissolved. Then, 6.8 g of NaOH was slowly added under stirring to neutralize the acrylic acid, with careful control of the addition rate to avoid localized overheating. After the solution cooled, 1.728 g of BC and 1.536 g of Fe_3_O_4_ were added, and the mixture was thoroughly stirred to ensure uniform dispersion of the solid particles. Next, 0.0576 g of NMBA was added as a crosslinking agent, followed by 0.0768 g of APS as an initiator after dissolution, with stirring until complete dissolution. The beaker containing the mixed reaction solution was placed in an oven at 70 °C and allowed to polymerize statically for 3 h until the system was fully cured into a black magnetic hydrogel. The beaker was then removed and cooled to room temperature. The hydrogel was taken out, washed by immersion in deionized water to remove unreacted impurities, and finally dried in a vacuum oven at 80 °C to constant weight. The dried hydrogel was cut into pieces, further dried, crushed, and passed through 30–50 mesh standard sieves to obtain the magnetic hydrogel product. The schematic diagram of the preparation process is shown in Figure 11.

The preparation processes of P(AA-AM)/SA, P(AA-AM)/SA-BC, and P(AA-AM)/SA-Fe_3_O_4_ hydrogels were similar to the procedure described above. The BC and Fe_3_O_4_ dosages were determined via single-factor optimization based on our previous studies, aiming to maximize adsorption capacity while maintaining sufficient magnetic responsiveness. It should be noted that the optimization in this study was performed using a single-factor approach; therefore, potential interactions among different parameters were not evaluated. Consequently, conclusions regarding optimal conditions may be limited in generalizability.

### 4.3. Swelling Property

To evaluate the swelling capacity, a pre-weighed dried sample was immersed in an excess of the test solution until equilibrium swelling was attained. The swollen gel was then separated from the residual liquid using a nylon mesh bag, and its mass was recorded. The equilibrium swelling ratio, SR (g/g), was calculated using Equation (6) [33]:

(6) $$\mathrm{SR}=\frac{M_{2}{-M}_{1}}{M_{1}}$$

where M_1_ and M_2_ denote the masses of the dry and swollen samples, respectively.

### 4.4. Adsorption Performance

For each adsorption test, 0.01 g of the sample was introduced into 50 mL of MB solution. Following a designated contact time, the supernatant was separated, and the residual MB concentration was measured using a TU-1900 dual-beam UV-vis spectrophotometer (Beijing Purkinje General Instrument Co., Ltd., Beijing, China) at 664 nm. The influence of initial pH on adsorption performance was investigated over a range of 2.0–12.0. Adsorption isotherms were constructed by varying the initial MB concentration from 10 to 70 mg/L. Regeneration of the MB-loaded adsorbent was achieved through desorption using 0.1 mol/L HCl. All experiments were carried out at room temperature. Unless otherwise specified, an initial MB concentration of 20 mg/L was used for all adsorption tests, with the exception of the adsorption isotherm studies. The adsorbed amount (Q, mg/g) and removal percentage (R, %) were derived from Equations (7) and (8), respectively [34]. Each experiment was conducted in triplicate, and the presented data represent the mean values.

(7) $$Q=\frac{{(C}_{0}-C_{e})V}{m}$$

(8) $$R(\%)=\frac{{(C}_{0}-C_{e})}{C_{0}}$$

where C_0_ and C_e_ are the MB concentrations (mg/L) at the initial stage and at equilibrium, respectively; m is the mass (g) of the dry hydrogel; and V represents the volume (L) of the solution.

### 4.5. Characterization

Fourier-transform infrared (FTIR) spectra were recorded on a NICOLETIN10 spectrometer (Thermo Fisher Scientific, Waltham, MA, USA) over the range of 4000–400 cm^−1^. Magnetic properties were characterized using a Quantum MPMS (SQUID) XL-7 vibrating sample magnetometer (VSM) (Quantum Design, San Diego, CA, USA). Scanning electron microscopy (SEM) imaging was conducted on an FEI QUANTA FEG450 instrument (FEI, Hillsboro, OR, USA). X-ray diffraction (XRD) patterns were collected with a Rigaku D/MaxII-2500VB2/PC X-ray diffractometer (Rigaku, Tokyo, Japan) under continuous scanning mode. Surface elemental composition was analyzed by X-ray photoelectron spectroscopy (XPS) on a Thermo ESCALAB 250XI spectrometer (Thermo Fisher Scientific, Waltham, MA, USA). The Brunauer–Emmett–Teller (BET) data of the samples were tested by a nitrogen adsorption instrument, AutoSorb iQ2 (Quantachrome, Boynton Beach, FL, USA).

## Figures and Tables

**Table 1:** Kinetic parameters.

Hydrogel	Pseudo-First Order Model			Pseudo-Second Order Model			Intra-Particle Diffusion Model		
	Q_e,cal_ (mg/g)	k_1_ (min^−1^)	R^2^	Q_e,cal_ (mg/g)	k_2_ (g/mg·min)	R^2^	K_1_	K_2_	K_3_
							(mg/g·min^1/2^)		
P(AA-AM)/SA-BC-Fe_3_O_4_	70.79	0.0505	0.9603	97.94	0.0011	0.9995	15.10	4.65	1.41

**Table 2:** Comparison of the adsorption capacity for MB with different adsorbents.

Adsorbents	Adsorption Conditions (Initial MB Concentration, pH)	Q (mg/g)	Reference
N-doped biochar	30 mg/L, 11.0	84.2	[28]
Hydrogel-biochar composite	20 mg/L, 11	23.53	[29]
HG/MTWBC nanocomposite	10 mg/L, 8	20.79	[30]
Fe_3_O_4_@TEB	50 mg/L, 6.8	113.35	[31]
β-cyclodextrin xanthate hydrogel/nickel oxide nanocomposites	100 mg/L, 7	310.55	[32]
P(AA-AM)/SA-BC-Fe_3_O_4_	20 mg/L, 10.0	94.3	This study

## Data Availability

All data generated or analyzed during this study are included in this published article.
